# The p12 Subunit Choreographs the Regulation and Functions of Two Forms of DNA Polymerase δ in Mammalian Cells

**DOI:** 10.3390/genes16020188

**Published:** 2025-02-03

**Authors:** Dazhong Xu, Selvaraj Ayyamperumal, Sufang Zhang, Jinjin Chen, Ernest Y. C. Lee, Marietta Y. W. T. Lee

**Affiliations:** 1 Department of Pathology, Microbiology and Immunology, New York Medical College, Valhalla, NY 10595, USA; sayyamperumal@nymc.edu (S.A.); jchen56@nymc.edu (J.C.); 2 Department of Biochemistry and Molecular Biology, New York Medical College, Valhalla, NY 10595, USA; zhangsufang@gmail.com (S.Z.); marietta_lee@nymc.edu (M.Y.W.T.L.)

**Keywords:** human DNA replication, DNA repair, DNA polymerase δ, cell cycle regulation, DNA damage response, homology-directed repair, tumorigenesis

## Abstract

There are two forms of DNA polymerase δ in human cells, Pol δ4 and Pol δ3, which differ based on their possession of the p12 subunit. The degradation of p12 has emerged as an important regulatory mechanism that controls the generation of Pol δ3. The underlying importance of this system lies in the altered enzymatic properties of the two forms of Pol δ engendered by the influence of p12. We briefly review how the balance of these two forms is regulated through the degradation of p12. We focus on the roles of Pol δ4, whose cellular functions are less well known. This is significant because recent studies show that this is the form engaged in the homology-dependent repair of double-strand breaks. We consider new horizons for future research into this system and their potential involvement in tumorigenesis.

## 1. Introduction

This review focuses on the nature and regulation of DNA polymerase δ activity in the mammalian system. Extensive studies on Pol δ, mainly of the human system, established that there are two forms of Pol δ: a tetrameric form (Pol δ4), consisting of the p125, p50, p68, and p12 subunits, and a trimeric form (Pol δ3) that lacks the p12 subunit [1,2,3,4]. The interconversion between the two forms is largely controlled by the degradation of p12 via the ubiquitin–proteasome system. The conditions that trigger degradation of p12 include cell cycle progression and the DNA damage response [5,6,7]. It is now clear that there are cellular regulatory circuits that deliver Pol δ3 on demand to serve its cellular functions.

The understanding of the cellular roles of these two forms of Pol δ requires knowledge of their enzymatic functions and the impacts of the binding of p12 to the p125 catalytic subunit. The underlying studies for the system are chronicled in more detailed, prior reviews [1,2,3,4]. In these, we identified key changes in enzymatic properties in the p12 subunit that underlie its behaviors in the more complex functions in DNA replication and repair. In this article, we briefly review our understanding of the two forms of Pol δ and discuss new directions of research in this area. We discuss how alterations of p12 may be involved in carcinogenic cells that may either impact the carcinogenic process or tumor cell resistance to chemotherapy.

## 2. Historical Perspective—The Subunit Structure of Human DNA Pol δ

Pol δ was the first eukaryotic DNA polymerase to be discovered that had a proofreading 3′ to 5′ exonuclease. This occurred in the late 1970s, well after the role of proofreading exonucleases had been established in procaryotes and bacteriophages [8,9,10]. In eukaryotes no evidence of such an enzyme existed, and Pol α was thought to be the main enzyme involved in DNA replication [11]. Pioneering studies for a mammalian DNA polymerase with an intrinsic 3′ to 5′ exonuclease were conducted by a group of investigators at the University of Miami in Florida, in rabbit bone marrow erythroid cells [12,13,14], calf thymus [15,16,17], and human placental tissues [18,19,20,21,22]. Isolation of the enzymatic activity resulted in the characterization of a dimeric enzyme, consisting of the p125 and p50 subunits. The p125 subunit was shown to harbor both the polymerase and 3′ to 5′ exonuclease catalytic sites. These earlier studies faced major difficulties because the amount of Pol δ in rabbit bone marrow, calf thymus, and human placenta is very small. It was believed that the enzyme was Pol α, contaminated with a very active exonuclease. A second DNA polymerase with intrinsic 3′ 5′ exonuclease activity was also isolated from different laboratories and named Pol ε [19,23,24,25,26]. The molecular cloning of the p125 subunit provided final proof of the existence of Pol δ [27,28,29]. The p125 subunit of the catalytic core (p125 and p50) was found to share a similarity greater than 60% with that of *Saccharomyces cerevisiae* (*S. cerevisiae*) Pol3 [28,29]. These studies dispelled the criticisms that “Pol δ” was an artifact arising from Pol α being contaminated with a very active exonuclease.

The third mammalian subunit of Pol δ was later identified as p66/p68 [30,31]. Lastly, the fourth mammalian subunit, p12, was identified [32]. The human heterotetrametric Pol δ4 was reconstituted by its expression in insect cells [33,34]. The functional roles of p12 and the arrangement of the four subunits were determined [35]. The study of Pol δ in *S. cerevisiae* showed that Pol δ was a trimeric enzyme with homologs of the p125, p50, and p68 subunits [36,37]. The trimeric *S. cerevisiae* Pol δ served as a model system for the study of eukaryotic Pol δ, which established it as the lagging-strand DNA polymerase and helped define the mechanisms for the synthesis of the Okazaki fragment [37,38,39]. The accepted model is that there is a division of labor between Pol δ and Pol ε for lagging- and leading-strand DNA replication, respectively [40,41]. Of note, Pol δ in fission yeast *S. pombe* possesses a fourth subunit, Cdm1, that has limited homology with p12 [42]. However, there is no evidence that it functions to regulate Pol δ activity like p12 does in the human system.

Another important contribution of these early studies was the discovery of the activation of Pol δ activity (p125/p50 core) from a distributive to a processive action [43]. This activation factor was found to be the proliferating cell nuclear antigen (PCNA), which has since been shown to be a platform for many DNA transactions [44]. p125, p68, and p12 have all been shown to bind to PCNA [1,2,3,4]. The p68 subunits of both human (p68) and yeasts (Pol32/Cdc27) possess PIP-boxes (PCNA-interacting protein) at their C-termini [36,38,45]. The p68 subunit has an elongated structure and is highly charged, suggesting that it is flexible and, thus, an ideal subunit for mediating PCNA interactions [46,47]. In some strains of *S. cerevisiae*, the Pol32 subunit is not essential, but Cdc27 is required for viability in *S. pombe* [46,47,48]. The p50 subunit also interacts with PCNA, although this interaction is much weaker [49,50]. An analysis of Pol δ4 enzymes in which the PIP-boxes of either p12 or p68 are mutated shows that both are required for full activity [35,51]. The p125 subunit binds to PCNA via the iron sulfur cluster in the CysB motif or the CysA zinc-binding segment located in its C-terminal domain [52,53,54].

## 3. Emergence of the Concept That Two Forms of Pol δ Are Involved in Human DNA Replication and Repair

Human Pol δ4 became the model for studying Pol δ in higher eukaryotes. Initial progress was slow due to limitations in terms of obtaining enough enzyme. This was later overcome by robust protein expression systems in insect cells [33,34,55]. These studies focused on the p68 and p12 subunits, noting that both were PCNA-binding proteins. Given that p12 may only exist in higher eukaryotes, there was a logical focus on the role of p12 in Pol δ functions based on the idea that it may regulate the balance between the trimeric (Pol δ3) and tetrameric (Pol δ4) forms of Pol δ.

### 3.1. Two Forms of Pol δ

The human Pol δ heterotetramer (Pol δ4) and its subassemblies have been successfully reconstituted in the baculovirus system [33,34]. Pol δ4 has also been expressed in the *Escherichia coli* (*E. coli*) system [56]. The use of the baculovirus expression system allowed for the preparation of highly purified Pol δ4 and its subassemblies for biochemical studies. Initial attempts at obtaining reproducible activities of Pol δ subassemblies, including that of the trimer lacking the p12 subunit, encountered difficulties [34]. This was traced to the instability of p12 during the isolation process; additionally, both the p68 and p12 subunits appeared to be more susceptible to proteases than the p125 and p50 subunits [55]. Immunoaffinity chromatography was used as a key component for the purification of Pol δ subassemblies [57]. The preparations of Pol δ4 and its subassemblies were monitored for the appropriate subunit stoichiometry because of the possibility of subunit loss during isolation [55]. These measures permitted extensive biochemical studies using various reconstitution assays to compare Pol δ4’s and Pol δ3’s ability to perform various DNA transactions [1,2,3,6,58].

**Figure 1:**
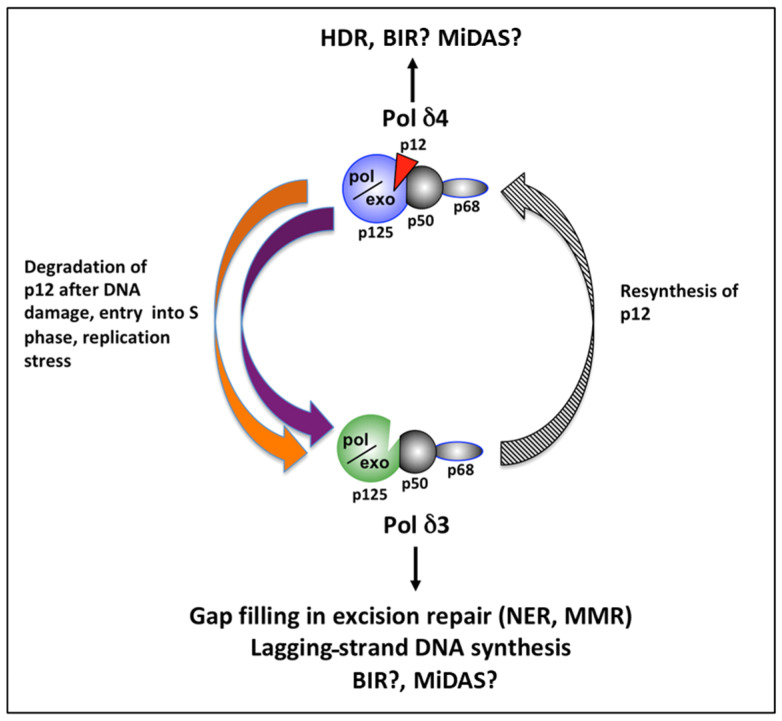
**The degradation of p12 controls the interplay between Pol δ4 and Pol δ3.** The left arm of the diagram gives a concise summary of our previous studies [1,2,3,4,58,59]. The regulated degradation of p12 is the key mechanism for the generation of Pol δ3. These studies also support an essential role for Pol δ3 in lagging-strand DNA synthesis and provide evidence regarding the differential roles of Pol δ4 in HDR. The delineation of the functions of Pol δ4 is still incomplete, and it may serve specific roles in BIR (break-induced replication) and MiDAS (mitotic DNA replication). The question marks are present to indicate that these are important areas for further study, especially regarding their comparative contributions to these processes.

The discovery that p12 undergoes transient degradation in response to DNA damage and during the S phase of the cell cycle led to the hypothesis that Pol δ4 and Pol δ3 serve distinct physiological roles in DNA damage and cell cycle progression and that their levels in cells are tightly regulated [4]. Pol δ4 is the predominant form in resting cells but is converted to the trimeric form by the controlled destruction of the p12 subunit. This occurs during the treatment of cells with UV, alkylating agents, and replication stress. p12 is degraded during the cell cycle, meaning that Pol δ3 becomes the primary form of Pol δ during the S phase and performs lagging-strand DNA synthesis during DNA replication. Availability of the p12 subunit switches Pol δ between its tetrameric (Pol δ4) and trimeric forms (Pol δ3) (Figure 1).

### 3.2. Interconversion of the Two Forms of Pol δ by Regulating p12 Availability

The existence of the two forms of Pol δ in cells is mainly determined by the level of p12. Available evidence suggests that the most important method to regulate the availability of p12 is protein degradation mediated by the ubiquitin–proteasome pathway. At least two ubiquitin E3 ligases, RNF8 and CRL4^Cdt2^, are involved in the degradation of p12 after DNA damage, while degradation during the S phase of the cell cycle is primarily regulated by CRL4^Cdt2^ [5,6].

RNF8 is a ubiquitin ligase that is activated in response to DNA damage and is involved in the repair of double-strand breaks (DSBs), particularly through the modification of histones and other DNA repair proteins and the recruitment of repair factors to the sites of DNA damage [60]. RNF8 was identified to be the E3 ubiquitin ligase of p12 using traditional protein biochemistry from Hela cells, subjecting the cell lysate to a series of affinity and gel filtration columns coupled with an in vitro ubiquitination assay, using p12 as the substrate, and employing mass spectrometry (LC-MS/MS) [5]. Follow-up experiments showed that p12 in cells lacking or with reduced RNF8 expression had a much longer half-life and higher levels after UV treatment compared to the control cells. These data indicate that RNF8 is a bona fide E3 ligase for p12 that mediates p12 degradation in response to UV [5]. The degradation of p12 by RNF8 in response to DNA damage leads to the conversion of Pol δ4 to Pol δ3, a form more suitable for gap-filling in excision repair (NER, MMR), associated with UV exposure [5].

The CRL4^Cdt2^ complex is a cullin ubiquitin ligase that plays a critical role in cell cycle regulation by controlling the degradation of cell cycle regulators and ensuring proper DNA replication. During the S phase, CRL4^Cdt2^ is responsible for targeting certain proteins for degradation to maintain the balance between DNA replication and repair [61,62]. Degradation of p12 in response to UV and other forms of DNA damage resembles that of the substrates of CRL4^Cdt2^, namely Cdt1, p21, and Set8. This observation prompted the logical question of whether p12 is also regulated by CRL4^Cdt2^. Sequence analysis revealed that the N-terminal region of p12 contains a well-defined PIP degron shared by all three known CRL4^Cdt2^ substrates [62]. Subsequent experiments showed that the depletion of subunits of CRL4^Cdt2^, Cul4, Cdt2, and DDB1, resulted in a prolonged half-life and the stabilization of p12 in response to UV, like in p21 and Cdt1 [6]. The critical function of CRL4^Cdt2^ in targeting Cdt1 for destruction to prevent re-replication during the S phase of the cell cycle inspired the examination of p12 expression during cell cycle progression. This led to the discovery that p12 expression is suppressed in the S phase via degradation mediated by CRL4^Cdt2^ [6]. The logic behind the CRL4^Cdt2^-mediated degradation of p12 in the S phase is likely to help convert Pol δ4 to Pol δ3, a form more adapted for lagging-strand synthesis during DNA replication [6,7].

UV- and hydroxyurea-triggered p12 degradation is blocked in ATR^−/−^ cells but not in ATM^−/−^ cells, demonstrating that p12 degradation is regulated by ATR but not ATM in response to these agents [63]. ATR is a central kinase in the DNA damage response, especially in response to replication stress. ATR activation is a crucial step in signaling DNA damage. It phosphorylates a variety of substrates involved in both cell cycle checkpoint control and DNA repair [64,65]. Thus, the degradation of p12 in response to DNA damage appears to be an integral part of the DNA damage response induced by these agents.

There remain many questions about which other ubiquitin ligases may participate in the regulation of p12 degradation in response to other potential cellular signals. On the other hand, additional complexities in the control of p12 degradation have emerged. A recent study has opened a new area for further research, viz., the discovery that p12 is a target of UCHL3, a ubiquitin C-terminal hydrolase in glioma stem cells [66]. It has been shown that UCHL3 induces radiation resistance and the acquisition of mesenchymal phenotypes by deubiquitinating p12 in glioma stem cells. This is of significant clinical relevance as the proneural-to-mesenchymal transition of glioma stem cells is associated with resistance to radiation therapy in patients with glioblastomas. The interpretation of these findings in the context of the two forms of Pol δ is consistent with HDR capacity for DSB repair mediated by increased Pol δ4 as a consequence of p12 stabilization (see Section 4). Another area for future investigation is related to the finding that p12 has also been shown to be a substrate of µ-calpain, a calcium-dependent cysteine protease involved in calcium-induced apoptosis [67]. Here, the concept that has emerged is that the sustained loss of p12 and the loss of HDR capacity could participate in the apoptotic response.

### 3.3. Functional Differences Between Pol δ3 and Pol δ4 That Are Conferred by p12

The rapid degradation of p12 in the S phase of the cell cycle and in response to DNA-damaging agents suggests that Pol δ3 and Pol δ4 may have distinct functional characteristics. Extensive evidence shows that these two forms have different enzymatic properties in lagging-strand DNA synthesis during DNA replication and DNA repair in response to DNA damage [1]. Compared to Pol δ4, Pol δ3 exhibits a lower tendency for bypass synthesis across secondary structures of DNA. Pol δ3 is also more efficient at proofreading than Pol δ4 and less likely to extend mismatched primers or misincorporate wrong nucleotides [68]. However, Pol δ3 does not exhibit strand displacement activity, which is important for the adaptation of Pol δ3 for Okazaki fragment synthesis and processing in cellular DNA replication [69]. With regard to DNA repair, Pol δ3 is less able to bypass DNA lesions generated by certain genotoxic agents, such as alkylating agents, reactive oxygen species, and UV [70]. However, Pol δ3 exhibits greater fidelity and adaptation for gap-filling in excision repair (nucleotide excision repair, mismatch repair, base excision repair) [69].

In contrast to Pol δ3, Pol δ4 has a better ability to overcome secondary structures during DNA replication [68]. However, this property may reduce the fidelity of repair as it is more likely to extend mismatched primers or misincorporate wrong nucleotides [68]. This could be the reason why Pol δ3 rather than Pol δ4 is the preferred form during the S phase of the cell cycle.

**Figure 2:**
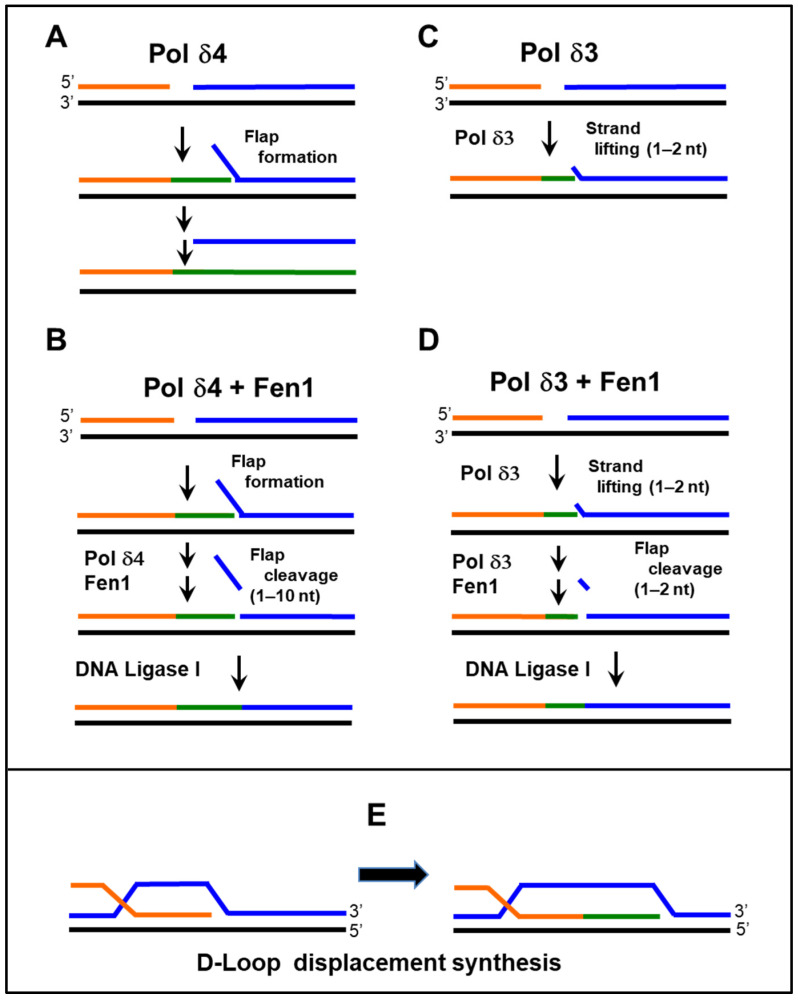
**Pol δ4 but not Pol δ3 exhibits stand displacement activity.** (**A**–**D**). Diagrammatic view of the ability of Pol δ4 and Pol δ3 to perform strand displacement on model oligonucleotide substrates and the processing of Okazaki fragments in concert with Fen 1 and DNA ligase using model oligonucleotide substrates [4,69]. (**E**). Displacement synthesis in a model D-loop (extension of invading strand). Pol δ4, but not Pol δ3, is predicted to be able to perform D-loop extension [58].

Human Pol δ4 possesses the ability for strand displacement, whereas Pol δ3 does not [69]. This emerged during studies of their abilities to perform Okazaki fragment processing in a reconstituted system [69]. It was clear that Pol δ4, but not Pol δ3, had the ability to generate a flap structure by strand displacement (Figure 2A–D). In contrast, yeast Pol δ, although lacking the p12 subunit, does have the strand displacement ability [71].

Pol δ4 is required for break-induced telomere synthesis [72,73]. However, evidence shows that Pol δ4 is not required for mitotic DNA synthesis (MiDAS), as the deletion of p12 does not affect this process [74].

### 3.4. Potential Division of Functions of the Two Forms of Pol δ—Expanding the Range of Pol δ-Specific Functions

It has been previously proposed that strand displacement is a requirement for D-loop extension in homologous recombination (HR), the extension of the invading strand, by DNA polymerases [75]. This would infer that Pol δ4, not Pol δ3, is involved in the homologous recombination process. Here, we focus our questions around whether Pol δ4 rather than Pol δ3 is involved in homology-directed repair, a key repair process for DSB repair. While there is evidence that Pol δ4 is competent in reconstitution assays for D-loop extension, the ability of Pol δ3 was not examined in said studies [76,77]. The direct comparison of Pol δ4 and Pol δ3 using in vitro assays showed that Pol δ3 was not effective [58]. Studies of p12 knockout cells in which the *POLD4* gene encoding p12 was ablated exhibit defective HDR, as well as heightened sensitivity to DNA-damaging agents and PARP inhibitors [2,59]. These p12 knockout cells are essentially null for the Pol δ4 enzyme and contain only Pol δ3. The fact that these cells were viable suggests that Pol δ3 is sufficient for cellular DNA replication. These results are supported by the observation that p12 knockout mice are normal and that tail-tip fibroblasts from these mice show no visible abnormality in proliferation and survival [78].

The findings discussed above represent a proof of principle for the division of functions between the two forms of Pol δ. However, they clearly represent the tip of an iceberg, given the many processes in which HR is involved. This also points to a major area for future studies, that of the exploration of the functions of Pol δ4. One potential avenue for expanding the repertoire of Pol δ4 is through interacting proteins that enhance its activities. We have demonstrated that the DHX9 helicase is able to stimulate D-loop synthesis by Pol δ4 [2,58]. However, Pol δ3 activity is not affected by DHX9 [58]. A more extensively studied role for the Pif1 helicase in break-induced replication has been established in the yeast system [79,80,81]. BIR involves the repair of broken chromosomes and generally involves the synthesis of long tracts of DNA. Much less is known about BIR in the mammalian system, although it has been shown to also require Pif1 [82]. Another area for future investigation is MiDAS (mitotic DNA synthesis), which is induced by replication stress and is known to require several HR factors and Pol δ activity [74,83].

In summary, the interconversion of Pol δ4 and Pol δ3 via p12 availability is functionally significant because Pol δ3, the trimeric form, is more important for normal DNA replication and excision repair, especially in response to replication stress. The conversion from Pol δ4 to Pol δ3 ensures that the polymerase complex is optimally adapted to the needs of the cell during times of DNA damage to maintain genome integrity. Pol δ4, the tetrameric form, is more specialized for HDR (Figure 1).

### 3.5. Mechanisms for Orchestrating the Division of Functions of Pol δ3 and Pol δ4

A question arises as to what other mechanisms help achieve the functional regulation of Pol δ3 and Pol δ4, since both may be present while the process of p12 degradation or resynthesis takes place. There may be situations where the presence of both forms may be deleterious, and their actions need to be compartmentalized. Several mechanisms may be operative. The first is the temporal separation of Pol δ3 and Pol δ4 during the cell cycle [1,2,3,4,84,85,86]. Without DNA damage stress, Pol δ3 only appears in the S phase, while Pol δ4 exists in the G1 and G2/M phases of the cell cycle [4,85,86]. Given that it is generally believed that a sister chromatid is required for HR to occur, Pol δ4’s functions in HR are likely restricted to late S to G2/M phases. Another route to the separation of function is through affinity for the PCNA/DNA primer template. In this regard, PDIP proteins (Pol Delta Interaction Proteins) could play a role, as exemplified by studies of PDIP46 [87]. PDIP46 facilitates Pol δ4 synthesis through model secondary structures but does not affect Pol δ3. PDIP46 could provide Pol δ4 with abilities to traverse hard-to-replicate regions of the genome. Pertinent to this discussion is that PDIP46 binds to p12. Thus, p12 could be a determinant for the selective recruitment of Pol δ4 but not Pol δ3. Another example (see above) is in the binding of DHX9 helicase to Pol δ4 to facilitate D-loop extension in HR [58]. Here, p12 could again function to allow the selective recruitment of a Pol δ4 partner protein that is required for a specific function.

## 4. Implications for Pol δ4 in Cancer

HDR deficiency is a major source of DNA abnormalities that lead to tumorigenesis, exemplified by the roles of BRCA1/2 in cancer [88]. The loss of HDR may force cells to repair double-strand DNA damage using the error-prone nonhomologous end-joining (NHEJ) repair mechanism, which can lead to mutations and allelic imbalance [88]. Given the significant role of Pol δ4 in HDR, it is conceivable that the loss of Pol δ4 because of p12 deficiency may lead to increased mutagenesis associated with DSB and produce mutational signatures resulting from the participation of other repair pathways or polymerases that may be more error-prone. Unlike BRCA1/2, which regulates multiple aspects of HR, Pol δ4 is needed at a defined locus in HR: D-loop extension. The mutational signatures due to the loss of Pol δ4 may or may not resemble those generated by BRCA1/2 deficiency, characterized by “Signature 3” (unbiased base substitutions) and large indels [89]. Despite the biochemical and cellular work pointing to a potential involvement of Pol δ4 in tumorigenesis, how Pol δ4 abnormalities contribute to cancer is largely unknown. The available evidence paints a rather complex picture.

Reduced levels of p12 were observed in human small-cell lung cancer (SCLC) and a fraction of non-small-cell lung cancer (NSCLC). Decreased expression is associated with a poor prognosis. Reduced expression of p12 appears to be a result of epigenetic suppression by the hypermethylation of histone H3 at lysine 9 coupled with the hypoacetylation of histone H3 [90]. Moreover, p12 depletion increases DNA damage and chromosomal instability. Interestingly, a delay in cell cycle progression at the G1-S boundary has also been observed in lung cancer cells. These effects have been reversed by restoring p12 expression [90].

In contrast to lung cancer, increased levels of p12 have been observed in glioblastoma and positively associated with cell proliferation and a suppressive immune microenvironment [91,92]. p12 expression is also positively linked to poor clinical outcomes in patients with glioblastoma and confers glioma stem cells (GSCs) a mesenchymal phenotype [66]. It has been established that the deubiquitinase UCHL3 binds and stabilizes p12 in GSCs. This study demonstrated that p12 directly increases the resilience of GSCs to radiation by increasing DNA repair via HDR and NHEJ. This study also showed that p12 expression was not affected by the inhibition of ATM and ATR in GSCs and had no effect on the cell cycle progression of GSCs [66].

A survey of the human cancer database revealed elevated p12 mRNA levels in most cancer types. However, reduced p12 mRNA expression was observed in several cancer types, including esophageal carcinoma, acute myeloid leukemia, small-cell lung cancer, tenosynovial giant cell tumor, uterine corpus endometrial carcinoma, and uterine carcinosarcoma [93]. This study also showed that the p12 isoform missing the entire exon 3 is expressed in renal carcinoma, thyroid carcinoma, pancreatic carcinoma, and low-grade glioma carcinomas, highlighting a potential regulatory role of p12 polymorphisms in cellular phenotypes [93]. However, a recent study showed that p12 knockout mice exhibit no change in tumorigenesis under the normal condition [78].

## 5. Future Prospectives

**Figure 3:**
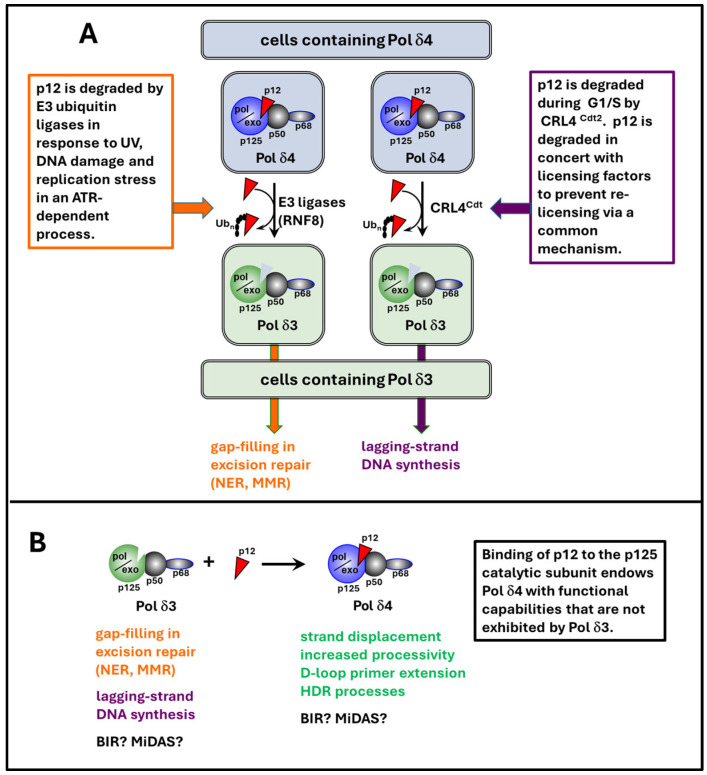
**The degradation of p12 in mammalian cells containing Pol δ4 to cells containing Pol δ3 choreographs their participation in DNA repair and replication processes.** (**A**) p12 degradation acts as a mechanism that dictates the two central functions in DNA replication and DNA repair. This diagram provides an overview of the cellular regulation of the degradation of p12, which converts the Pol δ4 tetramer to the Pol δ3 trimer. The removal of p12 leaves behind the Pol δ3 enzyme in vivo via the regulated destruction of the p12 subunit. Thus, cells containing Pol δ4 are converted to cells containing Pol δ3, a phenomenon which occurs under two scenarios. The first is the presence of genotoxic or replication stress (left-hand column). This occurs in an ATR-dependent manner by proteasomal degradation mediated by E3 ubiquitin ligases. Multiple E3 ubiquitin ligases may be involved, including RNF8 and CRL4^Cdt2^. This mechanism ensures that Pol δ3 is the operative form of Pol δ activity for gap-filling in excision repair (NER, MMR). The second trigger for p12 degradation (right-hand column) is integrally embedded in the cell cycle regulation of the initiation of DNA synthesis. Here, p12 degradation is ubiquitinylated and targeted for proteasomal degradation by CRL4^Cdt2^, which plays a key role in the destruction of licensing factors Cdt1, Set8, and p21 to prevent the re-licensing of the origins. Thus, cells in the S phase contain only Pol δ3, which is the lagging-strand polymerase in DNA replication. The system acts as a flip–flop switch between two cellular states where either Pol δ4 or Pol δ3 is the primary polymerase. This also constrains Pol δ4 from acting when Pol δ3’s function is operative. (**B**). The p12 subunit binds to the p125 subunit and thereby acts to modulate Pol δ4’s activity. This is of consequence because Pol δ4 possesses functions in DNA transactions that are not shared by Pol δ3. These functions represent “gain of function” attributes that expand the cellular repertoire of Pol δ activity. Notably, Pol δ4, but not Pol δ3, is the primary form engaged in the HDR repair of double-stranded DNA breaks. Thus, the involvement of p12/Pol δ4 in human DNA synthesis in homologous recombination is also subject to control by the p12 regulatory switch shown in (**A**).

Although much still needs to be learned, the biochemical function and biological significance of the two forms of Pol δ, Pol δ3 and Pol δ4, started to emerge after the discovery of the p12 subunit 24 years ago. It is apparent now that the two forms have different enzymatic properties that are adapted to serve different cellular functions. Pol δ3 is involved in gap-filling in several types of excision repair and is responsible for lagging-strand DNA synthesis, while Polδ4 is dispensable for DNA replication but is responsible for the key step of D-loop extension in HDR (Figure 3). The conversion of Polδ4 to Pol δ3 is triggered by UV, alkylating agents, and possibly other types of genotoxic stress.

Many questions remain to be answered. Among the central ones is the following: why does the system of Pol δ3-Pol δ4 interconversion exist? In a broader sense, what is the evolutionary rationale for higher eukaryotes to have this system, which is mostly absent in lower eukaryotes? As proposed above in Section 3.4 and Section 3.5, the answers most likely will emerge from the understanding of how different requirements for Pol δ functions are met and distributed between Pol δ3 and Pol δ4 in mammals. For example, information on a specific role of Pol δ4 in BIR and MiDAS is still incomplete. The pathological significance of abnormal p12 expression, which affects the level of Polδ4, also needs to be further explored. Of particular interest is its association with cancer, as the role of HDR defects in tumorigenesis is well established. The correlation of p12 expression and that of other subunits of Pol δ in cancer should also be studied in detail, as Pol δ4 does not function independently. The available information is very limited in this area.

Given the growing evidence that p12 expression varies greatly in different cancer types, the involvement of this protein in these cancer types may be complex and cancer-type dependent. For instance, the role of Pol δ4 at different stages of cancer development needs to be considered when determining the effect of p12 in tumorigenesis. In other words, the effect of Pol δ4 in non-transformed cells may be very different from that in cancer cells. This point is particularly relevant when evaluating the role of Pol δ4 in the neoplastic transformation of normal cells induced by genotoxic carcinogens. p12 expression may be epigenetically regulated at different stages of tumorigenesis to serve the needs of cells. Chronic vs. acute effects of carcinogens on p12 expression should also be considered. Thus, significant future research should focus on the role of Pol δ4 in the neoplastic transformation of normal cells and tumorigenesis in animal models in response to genotoxic carcinogens in order to fully appreciate the role of Pol δ4 in tumorigenesis.

The synthetic lethality revealed previously with p12 deletion and PARP1 inhibition resembles that of BRCA1/2 deficiency and PARP1 inhibition, which has led to therapeutic strategies based on synthetic lethality approaches and the concept of “BRCAness”, which allows targeting with drugs [94,95]. This finding not only helps define the involvement of Pol δ4 in HDR but also reveals a potential approach for therapeutic intervention. It also highlights the importance of studying this protein and associated biological processes. Future research efforts in this area are well justified and can be highly fruitful. As indicated in the Graphical Abstract, current studies also point to the probability that the control of p12 levels may be more complex than previously thought [96] (see Section 3.3). There now appear to be novel mechanisms that lead to increases in p12 levels, leading to an increased DSB repair capacity, which results in the promotion of tumor resistance to radiation. These studies also raise another important issue, in that the outcomes of alterations in p12 status, either in terms of increases or decreases, may be highly dependent on the individual cell or cancer type and exhibit different outcomes.
